# Societal and ethical issues in human biomonitoring – a view from science studies

**DOI:** 10.1186/1476-069X-7-S1-S10

**Published:** 2008-06-05

**Authors:** Susanne Bauer

**Affiliations:** 1 Medical Museion, Faculty of Health Sciences, University of Copenhagen, Fredericiagade 18, DK-1310 Copenhagen, Denmark

## Abstract

**Background:**

Human biomonitoring (HBM) has rapidly gained importance. In some epidemiological studies, the measurement and use of biomarkers of exposure, susceptibility and disease have replaced traditional environmental indicators. While in HBM, ethical issues have mostly been addressed in terms of informed consent and confidentiality, this paper maps out a larger array of societal issues from an epistemological perspective, i.e. bringing into focus the conditions of how and what is known in environmental health science.

**Methods:**

In order to analyse the effects of HBM and the shift towards biomarker research in the assessment of environmental pollution in a broader societal context, selected analytical frameworks of science studies are introduced. To develop the epistemological perspective, concepts from "biomedical platform sociology" and the notion of "epistemic cultures" and "thought styles" are applied to the research infrastructures of HBM. Further, concepts of "biocitizenship" and "civic epistemologies" are drawn upon as analytical tools to discuss the visions and promises of HBM as well as related ethical problematisations.

**Results:**

In human biomonitoring, two different epistemological cultures meet; these are environmental science with for instance pollution surveys and toxicological assessments on the one hand, and analytical epidemiology investigating the association between exposure and disease in probabilistic risk estimation on the other hand. The surveillance of exposure and dose via biomarkers as envisioned in HBM is shifting the site of exposure monitoring to the human body. Establishing an HBM platform faces not only the need to consider individual decision autonomy as an ethics issue, but also larger epistemological and societal questions, such as the mode of evidence demanded in science, policy and regulation.

**Conclusion:**

The shift of exposure monitoring towards the biosurveillance of human populations involves fundamental changes in the ways environment, health and disease are conceptualised; this may lead to an individualisation of responsibilities for health risks and preventive action. Attention to the conditions of scientific knowledge generation and to their broader societal context is critical in order to make HBM contribute to environmental justice.

## Background

The field of environmental health research is facing tremendous changes, as new biomarker techniques are developed in the biosciences: Biomarkers of exposure, susceptibility and effect have rapidly gained importance and feature prominent in many studies in environmental epidemiology. While environmental sciences have focused on monitoring exposure levels in soil, water and air, environmental exposures are increasingly measured via biological markers within the human body. In human biomonitoring (HBM), environmental field sciences meet concepts and methodologies from epidemiology and biomedicine.

Research into the use of biomarkers in environmental health is currently funded by many government agencies worldwide. The increasing importance of biomarkers in environmental health research is reflected in a number of seminal articles on HBM [1-6]. As part of national environment and health programmes, HBM initiatives have been pursued in several European countries from the 1980s onwards to survey the intake of anthropogenic chemicals [7-9]. More recently, larger biomonitoring projects were implemented in member states of the European Union to support standardised data collection and data processing. In the framework of its *Environment and Health Action Plan 2004–2010 *the European Commission initiated the development of a coherent approach to HBM [10,11].

At this moment of European harmonisation initiatives, reviewing the conceptual frameworks of HBM from a science studies perspective can inform the choices to be made both in research and in regulatory planning. In particular in view of the often stated need to fill knowledge gaps on the link between environment and health and the "ultimate goal [...] to develop an environment and health 'cause-effect framework'"  for policy [12], attention to the specific modes of knowledge production is important. To analyse conceptual frameworks and research practices, science studies can offer a range of tools, which include historical contextualisation, the ethnographic and microsociological study of single institutions, of particular laboratories or research networks and the analysis of interaction and co-production between science and society [13,14]. It is only recently that science studies accounts of environmental health research – at the intersection of public health and environmental science – begin to emerge. Mitman et al. [15] state that much of the most recent environmental health history has fallen between the cracks of environmental history, geography, history of science, medical history and science studies.

The human body became an object of quantitative science in the context of 19th century biometry and physical anthropology; metrics were applied to medicine and physiology as well as to all corners of science and society with avalanches of numerical data produced, including first censuses as large scale inventories of populations [16-18]. In the context of occupational medicine, techniques to measure the uptake of chemical substances directly in human body fluids were developed in the early 20^th ^century; measurements of lead and benzene metabolites were conducted by industry in the 1930s [8]. Large scale HBM was first and mainly practiced as workforce surveillance and later became part of regulatory requirements for some industrial sectors, mostly the chemical industry. Employers and industrial medicine have increasingly monitored workers' intake of hazardous chemicals and industrial medicine had an important influence on the development of the environmental movement in the second half of the 20th century [19]. Human biomonitoring can be situated in a tradition of occupational medicine that developed under the conditions of industrial hygiene as an applied science.

While the contemporary extension of HBM from occupational medicine to public health has often been framed in terms of an environmental justice agenda [20,21] aimed at reducing inequities in environmental health, monitoring chemicals among the general population also raises a host of societal and ethical issues, related to recruitment, participation and confidentiality, to the use, access and interpretation of data as well as to policy action.

This paper explores HBM and its conceptual frameworks and research practices that build on both environmental sciences and epidemiology; it uses science studies tools to broaden the ethics debate to include epistemological and societal aspects, such as the recent shift to biomarkers in exposure assessment and related conceptual changes in environmental health knowledge.

## Methods

This analysis of human biomonitoring (HBM) and its ethical problematisations focuses on the structures and modes of knowledge generation. By concentrating on epistemologies, this paper seeks to better understand the structure, the limitations and the stabilisation of knowledge in the field of environmental health sciences. For this purpose, an analysis based on a review of the scientific literature at the intersection of biomonitoring, epidemiology and environmental health from the late 1980s to present has been conducted. Analysed materials include published research papers on HBM in environmental science, epidemiology and human toxicology as well as review articles on the development of HBM and, in particular, on the use of biomarkers in epidemiological studies. In addition to the scientific literature on HBM as available in databases such as PubMed, reports and strategy papers for instance of the European Research Council have also been taken into account. The text analysis examined how HBM and biomarker research delineated their goals and scope, what kind of visions and promises become attached to the research endeavours and how ethical issues were problematised.

In order to explore the ethical and the broader societal issues that arise from population biosurveillance on a more conceptual level, this paper draws on epistemological approaches developed in science studies, i.e. "platform sociology" [22], "epistemic cultures" [23][24] and "thought styles" [25], as well as on frameworks that allow to explore the relations to citizenship concepts. While this paper spans from knowledge production to the ways this knowledge is taken up in policy and regulation, its point of departure is less in the debate on ethics principles, such as informed consent, confidentiality or the right to know or not to know. Rather, this paper takes a closer look at the broader societal aspects at stake in the emerging HBM platforms for knowledge production in environmental health.

Inspired by "platform sociology" [22], this analysis focuses on HBM research as an emerging infrastructure that incorporates both environmental and biomedical research traditions. Rather than requiring shared concepts, biomedical platforms are based on common technical and administrative infrastructures, which co-organise and also regulate the production of knowledge [22]. The standardised HBM platform is described as a distributed research system, which extends beyond environmental chemistry and toxicology laboratories to the health care systems, to include population health and society as a whole.

This paper analyses the different "epistemic cultures" of knowledge production that meet in environmental health sciences and in HBM in particular. The concept of "epistemic cultures" or "epistemological cultures" [23,24] brings into focus the arrangements and mechanisms of knowledge generation, the modes of knowing in different science communities and their criteria for what counts as scientific evidence. In order to juxtapose the "thought styles" [25] at work in HBM, environmental science and risk factor epidemiology will be introduced as distinct traditions for analytical purposes, although they can well overlap in practice.

The concepts of "biosociality" [26] and "biocitizenship" [27,28] will be employed to grasp the effects of HBM on the governance of populations [29] and the specific conditions of biosurveillance through HBM. To examine the role of HBM in the knowledge society, the notion of "biosociality", will be used to trace how social processes and networks are reconfigured along with bioscientific categories. "Biological citizenship" as used here denotes the shifts in the understandings of citizenship towards the active engagement in the emerging biosociety; this includes participation in health research as well as renegotiations of individual and group identities by actively drawing for instance on genetic categories.

The configurations at the science and society interface will be further explored by asking for "civic epistemologies", a term introduced by Jasanoff [30] in her comparative analysis of life sciences politics as a relationship between state, science and the public in the US and Europe. Civic epistemologies relate to the culturally specific, historically and politically grounded knowledge-ways and the process through which knowledge comes to be perceived as reliable [30].

Together, these perspectives make it possible to examine both the epistemological infrastructures and institutionalised practices by which knowledge is generated and deployed as a basis for political choices in society. This will enable a broader analysis of the bioethical debates in HBM and, more generally, of biomedical and epidemiological research.

## Results and discussion

### *Human biomonitoring (HBM)*: an emerging research platform

Human biomonitoring (HBM) has recently been defined as "the assessment of human exposure to an environmental chemical via the measurement of that chemical, its metabolite(s), or reaction product(s) in human blood, urine, milk, saliva, adipose or other tissue in individuals taken separately but generally taken together to constitute a population" [9].

Generating and interpreting HBM data involves a wide range of subdisciplines, for instance those of environmental chemistry, toxicology and epidemiology, with distinct experimental practices and conceptual frameworks. As will be elaborated in more detail below, the ways in which environment, health and risk are framed differ substantially between these disciplines; this affects the ways in which data are collected, made sense of and deployed in science and for policy decisions. What ties the different subdisciplines together in HBM, is the common infrastructure of data collecting and the joint aim of monitoring environmental determinants of health and disease. Processing individual data in an epidemiological framework, i.e. at levels of the population and subpopulations, is a key feature to the use of HBM data; this holds for descriptive analyses as well as for health risk assessments and aetiologic hypothesis testing for associations between environmental exposures and disease outcomes.

The European *Environment and Health Action Plan *stresses that "biomonitoring is not an automatic instrument, which can be considered in isolation, but has to be integrated with environmental monitoring, toxicological and ecotoxicological data and especially with the considerations related to analytical epidemiology" [11]. To account for the diversity of related disciplines, infrastructures and practices that are brought together in this common network, HBM can be described as extended "biomedical platform" in the sense that they comprise "material and discursive arrangements that act as the bench upon which conventions concerning the biological or normal are connected with conventions concerning the medical or the pathological" [22]. The HBM infrastructure encompasses databases as well as samples in biobanks, protocols for collecting practice and analytical routines. The emerging European HBM platform comprises a set of standardised methods and guidelines for deriving exposure limits and health risk estimates that enter policy decision-making.

With respect to the understanding and meaning of biomarkers, reviews often point to gaps in knowledge, data and validation that render interpretation difficult; for instance background and baseline values are not available for many ubiquitous environmental chemicals and their metabolites [5,31]. The envisaged goal of HBM studies, as stated in a 2005 summary report, is to establish baseline values and distributions of body burdens of environmental chemicals in the general population; foreseen next steps include identifying geographical clusters of elevated concentrations of biomarkers, studying time trends and evaluating the efficacy of regulatory action [5].

As a scientific resource, different visions and promises are attached to the HBM platform in environmental science and epidemiology, respectively: In environmental science, biomarkers of exposure are understood as extension to environmental monitoring, that will add important information in the surveillance of actual doses. For example, DNA adducts have for decades been researched as "biological dosimeters" that may "qualitatively indicate increased risk of cancer" [1]. Such biological dosimeters were anticipated to provide direct measures of environmental pollutants and early detection of exposure effects, yet the quantification and precision needed for deriving exposure limits proved difficult to achieve; new biomarkers are hoped to fulfil this function.

From the perspective of epidemiology, the value and potential of HBM is the methodological opportunity to reduce the bias introduced with exposure misclassification; reliance on biomarkers is therefore expected to strengthen epidemiologic risk assessments. Some biomarkers of exposure are hoped to enable direct measurement of actual combined exposures and to enable the determination of summary cumulative individual doses for epidemiological studies. However, biomarker measurements have also been reported to involve considerable imprecision beyond laboratory performance; the reliance on single parameters for a biomarker concentration is therefore problematic and such imprecision can underestimate the dose-related toxicity [32].

### Thought styles and epistemic cultures in HBM: environmental science and epidemiology

This section juxtaposes environmental science and (risk factor) epidemiology, as research fields that epitomise two main and different thought styles at work in contemporary environmental health science and in HBM in particular. Both environmental science and epidemiology represent empirical field sciences, yet they have developed within different disciplinary traditions and cultures of research. While environmental science performs descriptive mapping, monitoring and reduction of exposures to environmental chemicals, approaches of analytical epidemiology focus on disease aetiology and assess multiple associations between exposure and disease in statistical inference tests. In epidemiology, contestations over causality and the respective contributions of genetic, environmental, lifestyle or social "risk factors" are sorted out through statistical modelling of these determinants.

#### Environmental science

Environmental sciences – such as environmental chemistry and engineering – focus on exposure. Key practices of knowledge generation include developing monitoring systems of emission sources, modelling distribution patterns and pathways of environmental chemicals including their migration and uptake. In the thought style of environmental science, risk assessments model the dynamics of chemicals in environmental compartments, estimate exposure and doses at individual and collective levels; indicators are developed, monitored and related to regulatory frameworks with exposure limits that are based on toxicologic and epidemiologic assessments. Assessments from an environmental science perspective can include both human and ecosystem exposures. Different from epidemiological surveillance, this kind of monitoring does not necessarily imply immediate synthesis with health data and aetiologic hypotheses testing; rather it aims at precautionary risk management and exposure reduction.

In environmental sciences, the goal of "primary prevention" is the survey and control of environmental chemicals rather than aetiological research. In that it focuses on the environment as a societal issue with interventions on the level of regulation, the conceptual framework of the environmental science approach differs from the multiple individual risk factor concept used in epidemiologic assessments.

#### Epidemiology

No longer restricted to the study of infectious disease, epidemiology has often been described as the "basic science of public health". In his history of epidemiologic methods, Morabia tentatively characterises epidemiology as "population health aetiology" or "science of causation" [33]. Since the 1960s epidemiology has put forward an often pragmatic multifactorial approach to disease causation, which is dealt with through multivariate statistical modelling. The contemporary field comprises many subdisciplines, mostly named according to the exposures or the diseases studied – such as genetic, nutritional and radiation epidemiology and cancer, or cardiovascular epidemiology.

Environmental epidemiology investigates environmental conditions and their associations to disease outcomes with specific study designs and the related statistical techniques, for instance logistic regression modelling. While epidemiology largely originated in the field of biometrics, medical and social statistics and demography, the techniques of proof used in epidemiology have been widely applied from biomedicine to social sciences.

Epidemiological methods epitomise a certain thought style at work in biomedical and clinical research and becoming influential in public health. In particular with "evidence-based medicine" (EBM), epidemiologic methods have developed into a general technique of testing for association between determinants and outcomes. Rigorous epidemiologic study designs have become the methodological standard of proof which, like all biomedical research, environmental health issues must pass in order to achieve visibility. This epistemological shift to risk factor epidemiology as a powerful and robust technique has reconfigured the framework and conditions in which much of current environmental health research, including HBM, is taking place. The thought style of epidemiology shifts the framework towards conditions of proof developed in biomedical research. For HBM, this implies the requirement to conduct analytical studies, which demand a different set-up and data strategies than a surveillance and monitoring system.

#### HBM and the intersection of environmental health research

The databases and biobanks created in HBM have been envisioned as resources for aetiological studies of single and combined exposures as potentially causal agents. If however HBM data are collected mainly for the purpose of aetiologic research, this may lead to an emphasis of extending large-scale epidemiological studies (often initiated in other contexts) at the expense of studies into locally relevant exposure pathways. To obtain large numbers for aetiological investigations, existing multi-centre studies are supplemented with biomarker variables as surrogates for environmental exposure – while the exposure history and sources then remain difficult or impossible to trace.

To become articulated within the thought style and epistemic culture of analytical epidemiology, standards of proof developed in medical decision-making are increasingly drawn upon when discussing questions of causality in environmental health. The broad application of biomedical criteria to environmental health issues can be viewed as part of a more general "biomedicalisation", a term introduced, among others, by Clarke et al. [34] to denote the increasingly complex and multisited transformations of medicine through science and technology in the second half of the 20^th ^century, for instance with epidemiologic surveillance and risk assessment.

However, conceptual frameworks that strive to integrate the environmental perspective into the biomedical sciences have been developed in social epidemiology; with these frameworks of causation work, social epidemiologists aim at shifting the research focus from aetiological epidemiology and individualised risk estimates towards more societally modifiable factors to be addressed on the level of policy [35]. Eco-social frameworks of disease causation attempt to provide alternative models, using the concept of embodiment, in order to better account for the complex interaction with social and environmental factors [36]. In the development of an integrated HBM platform, awareness of the conceptual framework, of the level of data production and of the relations between biomarkers and pathways of exposure as well as the conditions of knowledge and their social dimensions will be important.

#### Epistemological issues – what does count as evidence?

Software platforms for multi-layered data mapping of environmental and social factors, e.g. geographic information systems as envisaged for HBM data platforms, are key tools to map data onto spatial or temporal grids both in environmental research and epidemiology. Environmental sciences draw extensively on mapping techniques in the tradition of atlases – for instance in environmental atlases or, in descriptive epidemiology, cancer atlases. Such public health maps are often traced back to John Snow's paradigmatic epidemiological study on cholera and contaminated water in 19th century London.

Different from this type of descriptive accounts of aggregated data, analytical epidemiology is about aetiological hypotheses testing and statistical inference based on datasets with individual level records that allow control for confounders. Evidence-based medicine sorts out epidemiologic results by study design – from bench research and singular expert opinions (which are understood as belonging to the lowest level of evidence) to case-control studies, ecological studies, cohort studies (considered intermediate levels) to randomised clinical trials (RCTs) and culminating in meta-analyses of RCTs (considered the highest levels of evidence) [37]. Epidemiological study designs are conceived within a hierarchy depending on the degree of evidence attributed to the study design.

Table 1 summarises a system of evidence hierarchies published by the Oxford Centre for Evidence-Based Medicine (EBM) [37]. In clinical epidemiology, EBM has developed standards on how to evaluate scientific studies for medical decision-making. In the context of clinical medicine, this objectification of medical knowledge through EBM was initiated as part of a movement [38], opposed to the prevailing clinical hierarchies. The evidence-based approach has been discussed in many fields beyond medicine, for instance in evidence-based policy or environmental management [39]. As the table shows, a substantial part of approaches in environmental epidemiology does not reach beyond an intermediate level of evidence in this hierarchy. What has been conceived as an innovation in hospital medicine, can bear different implications in other domains, including HBM. The regulation and hierarchisation of scientific evidence, which privileges the RCT followed by cohort studies as the highest mode of evidence, can seldom be met in environmental health studies. With the recommendations for EBM, geographic mapping can hardly provide sufficient evidence for an environmental policy decision. For environmental issues, this mode of evidence has significant repercussions to concepts of precautionary action.

**Table 1:** Evidence hierarchies for questions of therapy/prevention and aetiology/harm (abridged) [37]

**Level of evidence**	**Study type**
1a	Systematic reviews of randomized controlled trials
1b	Individual randomized controlled trials
1c	All or none randomized controlled trials
2a	Systematic reviews of cohort studies
2b	Individual cohort study or low quality randomized controlled trials
2c	'Outcomes' Research; ecological studies
3a	Systematic review of case-control studies
3b	Individual case-control study
4	Case-series (and poor quality cohort and case-control studies)
5	Expert opinion without explicit critical appraisal, or based on physiology, bench research or 'first principles'

On the one hand, HBM holds the promise to better assess actual doses and is therefore hoped to advance environmental health studies within this evidence hierarchy and provide more visibility as to environmental factors in analytical epidemiology. On the other hand, the contextual value of descriptive studies and mapping in environmental sciences is often neglected, when the main reference point resides in this formalised order of evidence hierarchies. Furthermore, error ranges and uncertainties in the interpretation of biomarkers are substantial, in part as biomarker concentrations vary between individuals and are often not persistent over time; misclassification due to error in exposure measurement may lead to underestimation of the effect [31,40]. More general criticisms have pointed to the limits of epidemiology in particular in environmental health, concluding that, given the uncertainties involved, a precautionary approach is needed when it comes to environmental health risks [41-43].

At stake in the development of an evidence-based environmental science, as envisioned also by some environmental science initiatives [39], are precisely the modes of proof and evidence in environmental health sciences. To meet the conditions of proof, individual-level data and, more importantly, randomised assignment of interventions are called for. The following section will turn to the example of an ethics case that evolved around the pursuit of a rigorous experimental design in environmental health research.

### Ethical problematisations as societal issues

#### Environmental epidemiology, "experimental" studies and regimes of imperceptibility

In clinical and epidemiological research practice, ethical issues are often solely dealt with at the level of participation, e.g. in terms of informed consent, confidentiality, the right to end participation at any time, under reference to individual decision autonomy [44]. Yet, another aspect evolves around the issue of what kind of evidence is demanded for environmental health measures. A much debated example of an environmental health ethics case is the lead paint abatement study conducted at the Kennedy Krieger Institute affiliated with Johns Hopkins University in Baltimore (USA) [45]. Lawsuits and a major ethics controversy accompanied this intervention study, in which public health researchers investigated the effects of abatement procedures among children in a disadvantaged housing area in Baltimore. The intervention trial was designed to study whether less expensive abatement methods had the same effects as to reducing elevated blood lead levels.

The study raised a debate on the ethics of non-therapeutic research, in particular with children. Parents sued the responsible institute because they weren't adequately informed about the aim of the study and potential risks to their children. The judge put forward the argument that the children were intentionally put at risk and used as "measurement tools" in the study [46]. In the debate that followed, public health researchers argued for a pragmatic approach to ethics, since the proof for the effect of a cheaper abatement method could in future benefit large numbers of disadvantaged children, when informing environmental policies in a situation of scarce resources [47]. Others maintained that the experimental trial was unethical, e.g. since landlords were deliberately encouraged to rent homes with known lead contamination to families with children; research aimed at saving money and the cost-benefit reasoning was seen as problematic in itself [46]. This mode of public health research was criticised for being more concerned with the quality of the "experiment" than with the actual benefit or harm to participating subjects.

Stating the lack of sufficient evidence from rigorous epidemiological studies is problematic for chemicals with well documented toxic effects such as lead. It has been viewed as unethical to conduct environmental health studies at the level of best evidence (randomised trial examining cheaper interventions) instead of providing the best abatement, similar as it is widely considered unethical to continue a clinical trial after it has provided evidence for the superiority of a treatment. Both the use of EBM in the allocation of resources [48,49] and the principle of equipoise in clinical trials [50-53] have been subject to intense debate in the past two decades.

While the researchers strived to generate knowledge at the highest possible level of evidence according to the standards of the discipline, it was the experimental design to achieve this that resulted in ethical controversy. This was particularly sensitive because the tested hypothesis was whether cheaper abatement was as efficient as more expensive abatement. Yet in another twist to the ethics debate, some epidemiologists held that it would be unethical not to conduct such studies, because it would preclude the allocation of resources to more people. At stake in these ethical problematisations were the terms of proof and the level of evidence that is considered justifying the allocation of resources, when the distribution of resources in environmental health is decided upon. These involve trade-offs, which in this case centred on cost aspects rather than on environmental risk.

The perceptibility of environmental risk in standard epidemiological methods is rather narrow, due to long time lags for late effects and multiple interactions that are often difficult to investigate. This also holds on an epistemological level – due to the asymmetries in the ways false positives and false negatives are treated in the default procedures of hypotheses testing in both epidemiology and toxicology: the standard methodological tradition gives the reduction of type I error with 95% significance levels more emphasis than avoiding false negatives by securing sufficient power [42,43]. Therefore, both the formulation of the research question and the conditions under which the null hypothesis (that there is no influence of the exposure to disease) can be rejected, for instance the confidence level in relation to the power of a study, are also matters of epistemological politics. For the methodological limitations and the contingent invisibilities of environmental health risks, Murphy introduces the notion of "regimes of imperceptibility" [54]. The perceptibility of environmental health risks as scientific and societal issues depends on how the standards of proof are set.

In view of the ethical issues arising in prospective observational studies that would evaluate preventive interventions, the implications of the very frameworks for evidence and proof for science and policy action need to be discussed; this cannot be resolved by science alone; they require a broad debate on the conditions of proof requested for action on a societal level. To account for fields where no RCTs are possible, a protocol for observational studies [55] has been developed, similar to the idea of the Cochrane collaboration; for environmental health, these evidence criteria require further reflection and elaboration. The perceptibility of environmental health issues will depend on how the concept of evidence is framed and on how the burden of proof for environmental risks is distributed in society.

#### Biosocial shifts: from exposure assessment to markers of risk and susceptibility

This paper has so far mostly addressed the use of biomarkers of exposure in HBM. However the *European Environment and Health Action Plan *envisions a broader understanding of HBM as the "monitoring activities in human beings, using biomarkers, that focus on environmental exposures, diseases and/or disorders and genetic susceptibility, and their potential relationships" [11]. Extending beyond markers of exposure, biomarkers of disease and genetic susceptibility raise more and different ethical and societal issues; these have far reaching implications for our understanding of environmental health as well as for policy and regulation.

In a 2004 report, the *Genomics Task Force *of the *US Environmental Protection Agency *(EPA) envisioned how individual genetic susceptibility could be integrated into future policy and regulation. "If, for example, a genomics study were to identify a susceptible population at risk due to exposure to a contaminant at a Superfund site through a correlation of genomic analysis of local populations and measured or expected exposure levels, the Agency might choose to reduce the RfD/RfC [inhalation reference concentration/oral reference dose] value and propose more strict remediation measures. [...] Use of new genomics tools could, however, limit the extent of remediation measures by more accurately predicting the potential for exposure of the sensitive population. Thus, genomics tools may play a key role in determining intensity and extent of clean-up practices and have large implications for time and cost of such procedures" [56]. Thus, concepts and management principles of environmental and occupational health could change tremendously towards a differential model of protection against environmental pollutants: In this US report, individual and collective susceptibility profiles are envisioned to co-determine the extent of clean-up. This may significantly alter the notion of (environmental) equity and justice, as well as the related regulatory politics.

On the level of societal effects, the rise of biomarker assessments leads to more individualised accounts of health risks, as it is measured via biomarkers together with individual susceptibility. This trend can be viewed as further fostering "biomedicalisation" [34] of environmental health, as it shifts not only the site of monitoring to the individuals, but also envisions environmental management and decisions on clean-up as dependent on the genetic susceptibility of those exposed to environmental pollution.

While biomarker research is hoped to provide more precise assessment of combined exposures, it cannot offer information on exposure sources and histories; as biomarkers measure individual uptake, they often remain unspecific with respect to exposure. As the *European Environment and Health Action Plan *states, "responses can then focus either on exposure by reduction or elimination of the pollutant or on health effects by prevention, early identification or stopping the progression of disease. The responses may also concentrate on the individual encouraging a behavioural change or medical intervention"[11]. This statement extends the responses to the individual; these can include optimising and reducing other risk factors. It is likely that the paradigm of risk factor epidemiology will rather result in measures on the level of individual behaviour than in exposure reduction. The risk factor approach has been extremely successful in western societies, as it represents a framework open to a host of potential determinants and therefore easily perceived as neutral. Risk factor epidemiology has proved successful in formally integrating multifactorial causation and at the same time fostering the primacy of individual decision autonomy, which constitutes health as an issue to act upon individually [57].

Supplementing biomarkers of exposure by markers of genetic susceptibility tends to further frame the effects of exposure in terms of individual biology and, potentially, individual responsibility. It is the mobilisation into individual risk management that can be seen as part of an emerging "biological citizenship" [26,27], as society, environmental policies and the health care system appear reconfigured according to bioscientific categories.

#### Human biosurveillance and civic epistemologies

The human biosurveillance of environmental chemicals involves many stakeholders – government, industry, researchers and non-governmental organisations and individuals. In the European Union (EU), HBM is currently developed with the aim of providing integrated health impact assessments by linkage to other environmental and health data. Building a common platform is envisioned in two steps: First, available data will be linked at an aggregate level, e.g. by reference to geographic units. Clusters of elevated exposure, dose and disease prevalence can then be identified with geographic information systems as a descriptive tool. In a second step, individual-level data will be used to test hypotheses by linkage to established cohort studies or by the design of new follow-up studies [12].

HBM has been evoked as a tool to monitor environmental equity, in terms of detecting areas or population groups with elevated exposures via biomarkers. The European framework suggests biomonitoring as confirmatory when (environmental) monitoring indicates exposure of a population: "Biomonitoring can then verify the actual exposure and so provide better evidence for guiding appropriate responses" [11]. Although the report stresses that biomonitoring should complement exposure assessment as part of an integrated framework, the envisioned confirmatory use of HBM might result in transforming the standards of proof demanded in environmental health; thereby the indications to act upon exposures might be shifting to the level of biomarkers.

Working on a standardised approach to support HBM, bottom-up approaches have been drawn upon as well; these survey actual practices, ethical frameworks, interpretation of findings and policy issues [58,59]. The current harmonisation efforts have revealed differences in regulatory concepts and practices of the member states; these differences depend on whether they are part of occupational or environmental health contexts. When assessing inequalities in environmental exposures within and between regions, this process faces not only different methodologies but also different traditions of environmental monitoring, health surveillance and medical record keeping routines, which both reflect and produce different local "civic epistemologies" [30].

As to harmonisation effort in a different field – that of social statistics – input harmonisation and output harmonisation have been discussed as possible instruments to secure data comparability [60]. Here, input harmonisation has been understood as somewhat ideal data recording system in a common political space, where data are collected in identical formats. However this wouldn't allow for regionally specific studies, because the context-specific variables would no longer be available and locally meaningful research may be precluded. Output harmonisation secures comparability, yet allows for diversity as to the variables that are collected. This experience from social statistics and awareness of related implications for civic epistemology could prove instrumental in keeping the HBM system open enough to address locally relevant environmental health inequities.

## Conclusion

The shift of exposure monitoring towards the biosurveillance of human populations involves fundamental changes in the ways environment, health and disease are conceptualised; this potentially leads to an individualisation of responsibilities for health risks and preventive action. In developing the HBM platform as an infrastructure to secure environmental health equity, awareness of the epistemological and societal frameworks both from environmental science and epidemiology will be critical. Science studies tools can help understand the epistemological and the societal stakes in the field of environmental health. Accounting for the different thought styles at work in HBM can be of relevance in the decisions to be made during implementation and validation as well as in the translation of data to environmental policy. Attention to the societal dimensions of biosurveillance is crucial, when it comes to developing more participatory approaches and applying HBM data in regulatory politics.

## Competing interests

The author declares that they have no competing interests.
